# MYC in Regulating Immunity: Metabolism and Beyond

**DOI:** 10.3390/genes8030088

**Published:** 2017-02-24

**Authors:** J.N. Rashida Gnanaprakasam, Ruoning Wang

**Affiliations:** Center for Childhood Cancer & Blood Diseases, Hematology/Oncology & BMT. The research Institute at Nationwide Children’s Hospital, Ohio State University, Columbus, OH, USA, 43205; josephin.gnanaprakasam@nationwidechildrens.org

**Keywords:** MYC, immunity, metabolism

## Abstract

Myelocytomatosis oncogene (MYC) family members, including cellular MYC (c-Myc), neuroblastoma derived MYC (MYCN), and lung carcinoma derived MYC (MYCL), have all been implicated as key oncogenic drivers in a broad range of human cancers. Beyond cancer, MYC plays an important role in other physiological and pathological processes, namely immunity and immunological diseases. MYC largely functions as a transcription factor that promotes the expression of numerous target genes to coordinate death, proliferation, and metabolism at the cellular, tissue, and organismal levels. It has been shown that the expression of MYC family members is tightly regulated in immune cells during development or upon immune stimulations. Emerging evidence suggests that MYC family members play essential roles in regulating the development, differentiation and activation of immune cells. Through driving the expression of a broad range of metabolic genes in immune cells, MYC family members coordinate metabolic programs to support immune functions. Here, we discuss our understanding of MYC biology in immune system and how modulation of MYC impacts immune metabolism and responses.

## 1. Introduction

Cellular myelocytomatosis oncogene (*C-MYC*) (referred to here as *MYC)* was the first Myc family member found in the human genome and was originally identified as a cellular homologue of the avian myelocytomatosis retroviral oncogene (*v-Myc*) [1,2]. Since that time, extensive research has implicated it in regulating cellular growth, cell cycle, cell metabolism, and cell death. All of which collectively contribute to its oncogenic function [3,4,5,6,7]. Physiologically, MYC gene is expressed during embryogenesis and in tissues that are linked with high proliferation. Concomitant with promoting cell growth and proliferation, MYC inhibits terminal differentiation of most cell types and sensitizes cells to apoptosis [8,9]. Oncogenic activation of MYC can be achieved through gene amplification, chromosomal translocation, or mutation. In addition to direct genetic derangement, many other oncogenes or tumor suppressors can alter the expression of MYC [10,11,12,13]. As such, MYC overexpression is a characteristic hallmark of a broad spectrum of cancers and can directly lead to malignant transformation in several cancer types [4]. Similarly, deregulation of two other MYC family members, MYCN (also referred as N-Myc) and MYCL1 (also referred as L-Myc), has also been revealed in human neuroblastoma, breast cancer, lung cancer, and many other cancers [9,10,11,14,15,16].

MYC is a basic-helix–loop–helix leucine-zipper (bHLHZip) transcription factor that forms a heterodimer with Myc associated factor X (MAX), binds the E-box element CACGTG, and drives gene expression. Although MYC appears to be dedicated to MAX, MYC can also dimerize with itself [17]. In addition, MAX binds to members of the MAX dimerization protein (MXD) family through the HLHZip region and these interactions can also indirectly regulate MYC activity. Importantly, mutations in the MYC bHLHZip domain completely abolish MYC’s functions in cells. Several lines of evidence also suggest that binding affinity for its partners and sub-nuclear localization patterns impact MYC’s biological functions [1,17,18,19]. MXD (protein family transcriptional repressors like MXD1, MXI1, MXD3, and MXD4 are associated with terminal differentiation, inhibition of cell-cycle progression, and tumor suppression [8,20]. MYC functions can be antagonized by the interaction between MXD/MXI1 bHLHZip transcription factor proteins, which in turn suppress MYC mediated transformation and tumor growth [11,20].

## 2. MYC in Immunity 

**Figure 1:**
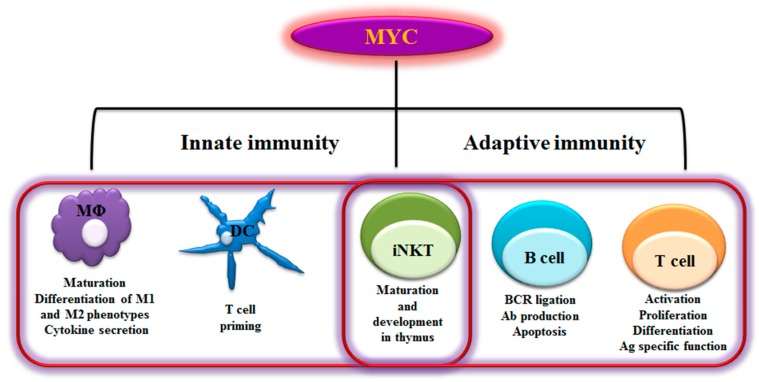
Myelocytomatosis oncogene (MYC)-dependent Immunity. As schematically shown here, MYC plays a role in regulating a range of innate and adaptive immune cells. MYC is a key transcription factor that regulates immune cell maturation, development, proliferation and activation. MΦ- Macrophages; DC- Dendritic cells; iNKT- invariant Natural killer T cells; BCR- B cell receptor; Ab- Antibody; Ag- Antigen; M1- Classically activated macrophages; M2- alternatively activated macrophages-

Immune response is an evolutionarily conserved process that protects multicellular organism from pathogens. The immune system is comprised of a variety of cells and molecules that are capable of identifying and eliminating foreign invaders, but not of self-tissue molecules. In order to do that, they have a powerful capability for learning, memory, and pattern recognition [21,22,23]. The immune responses to foreign antigens rely on both innate and adaptive immune components. The innate immune response is largely mediated by macrophages, dendritic cells, natural killer cells, monocytes, neutrophils, complement proteins, act together in a dynamic network to provide immediate host defense. Adaptive immune response consists of antigen-specific reactions through the proliferative burst and functional polarization of T lymphocytes and B lymphocytes. Innate immune response is rapid and short, but sometimes damages normal tissues through lack of specificity, whereas the adaptive response is long and precise, and takes several days to develop. The invading pathogens of vertebrates often rapidly reproduce and spread. As an essential transcription factor, MYC regulates numerous genes in both innate and adaptive immune cells and directs their activation, proliferation, polarization, and subsequent functional events elicited by these cells (Figure 1).

### 2.1 B Lymphocytes

B lymphocytes are bone marrow derived lymphocytes that express different cell surface immunoglobulin (Ig) receptors recognizing specific antigenic epitopes. B cells are activated by the binding of ligand to the B cell receptor (BCR), which further initiates numerous intracellular signaling cascades, and also triggers antigen presentation to elicit efficient immune functions [24,25]. MYC, as an important component of the BCR mediated transcriptional network, plays an important role in maintaining B cell homeostasis through regulating immature and mature B cell growth, differentiation, and apoptosis [26]. MYC by itself regulated by c-Rel/Nuclear Factor Kappa B (NF-κB), phosphoinositide 3-kinase (PI3K), protein kinase C theta (PKCθ), and nuclear factor of activated T cells (NFAT) signaling pathways in B cells. B cells with impaired c-Rel/NF-κB dimer fail to upregulate myc protein and lead to cell growth defect in mature B cells following mitogenic stimulations [27,28]. In addition, the expression of myc protein is required to elevate the intracellular Ca2+ level and activate NFAT, contributing to the B cell development [29,30].

MYC acts as a critical regulator of both proliferation and apoptosis upon the activation of BCR signaling. Deletion of MYC results in a gross phenotypic change and the transcriptional perturbation of numerous genes [26]. B cells lacking MYC show a poor response towards BCR ligation and resistance to apoptosis [26]. While IL-4 and anti CD40 activation can execute the early signaling activation of B cells in MYC deficient B cells, these cells fail to complete subsequent downstream activation events and display an impaired mitogenic response [31].

Downregulation of MYC expression in B cells also impacts the induction of apoptosis. MYC deficient B cells show a normal expression of activation markers, but are more resistant to CD95 and staurosporine induced cell death, indicating that MYC is an essential element in sensitizing cell death stimulus in B lymphocytes [32]. Additionally, MYC deficiency impairs B cell proliferation by increasing the expression of p27 that suppresses cyclin-dependent kinase-2 (Cdk2) activities, resulting in pRB hypo-phosphorylation and cell cycle arrest at the G0/G1 [31]. MYC dependent cell growth is associated with an increase of protein synthesis but independent of the cell cycle phase, indicating that MYC may coordinate the expression of growth related genes in response to mitogen signals [30]. However, deregulation of MYC expression causes B cell lymphoma through enhancing cell growth, promoting unrestrained cell division and strengthening pro-survival signaling. During B cell development, upregulation of myc protein under the control of Ig heavy chain enhancer results in the increase in cell size and malignant transformation [30,31]. 

### 2.2 T Lymphocytes

T cell progenitors that are derived from hematopoietic stem cells in the bone marrow migrate to the thymus to complete their maturation and their lineage determination into CD4 or CD8 T cells. Mature naïve T cells circulate through blood and the lymphatic system, and migrate to the periphery where they primarily reside in secondary lymphoid organs such as the lymph nodes and spleen [33,34]. As a key player of adaptive immunity, mature T cells can recognize antigen and rapidly transit from a naïve to an active state that is concomitant with cell growth, proliferation, and differentiation into functional groups. Following the phase of pathogen clearance, the majority of T cells will undergo apoptosis. The remaining small number of cells differentiate in to memory subset, which responds more effectively upon encountering the same pathogen [33,35].

MYC is an essential factor that provides the proliferative and pro-survival signals upon the engagement of T cell receptor (TCR) and also cytokine IL-2 mediated signaling. Downstream of TCR and Interleukin (IL)-2 signaling, the activation of protein kinases C (PKC) and increased intracellular calcium, drive the transcriptional induction of MYC [36,37]. Moreover, it has been shown that 80% of the genes repressed by MXD1 have been found to be induced by MYC, postulating that cell proliferation may be controlled by the relative levels of MYC and MXD-1 [38]. Additionally, expression of myc protein is required for proliferation, survival, and differentiation of immature thymocytes following the activation of pre-TCR and instructing them to the αβT-cell lineage efficiently [39]. Myc protein influences proliferation at the pre-TCR checkpoint by downregulating the expression of cell-cycle inhibitors and p27Kip, along with growth arrest and DNA damage-inducible 45 (Gadd45), rather than by directly affecting the expression of the cyclins or other cell cycle promoters [39]. Conversely, deregulation of MYC, either by MYC gene translocation or by Notch-driven MYC overexpression, impairs T cell development and induces T cell acute lymphoblastic leukemia (T-ALL) [40,41,42]. In addition, MYC is required for the development of a subset of intraepithelial lymphocytes (IELs) that express CD8αα homo dimers in the gut [43]. Inactivation of MYC in thymocytes leads to dramatic reduction of CD8αα IELs by inducing apoptosis, suppressing differentiation, and blocking migration of CD8αα TCRαβ IELs from the thymus to the gut where IL-15 provides a pro-survival and maturation signal to these cells. Specifically, MYC downregulation decreases the expression of IL-15R subunits and significantly lowers the expression of the anti-apoptotic protein Bcl-2 in the residual CD8αα TCRαβ IELs [43]. 

Mature naïve T cells are in the quiescent stage and express low levels of MYC. Upon the engagement of antigen and costimulatory molecules, MYC is rapidly induced as one of the immediate early-response genes and functions as a universal amplifier of gene expression increasing output at all active promoters [44]. The activation of PKC and intracellular calcium-dependent signaling drives early expression of MYC and promotes the production of pro-inflammatory cytokines such as IL-15 and IL-2, the latter of which induces a positive feedback loop through the IL-2 receptor and the janus kinase/signal transducers and activators of transcription (JAK-STAT) signaling pathway to sustain MYC expression throughout the proliferation phase [36]. A sustained MYC-dependent effector T cell function also requires activating enhancer binding protein 4 (AP4), which is a transcription factor regulated by IL-2R signaling and MYC [45]. AP4 sustains the proliferation, differentiation and cellular activity of antigen-specific CD8+ T cells through regulating a substantial proportion of genes that are targets of MYC. As such, AP4 deficient CD8+ T cells fail to maintain the transcription of a broad range of MYC-dependent targets, indicating that AP4 is required for a sustained expression of MYC targets. Thus, AP4 deficient mice display enhanced susceptibility to virus infections [45].

### 2.3 NKT Cells

Natural killer T cells (NKT cells) are a heterogeneous group of CD1d-restricted immunoregulatory lymphocytes that share properties of both T cells and natural killer cells. A dominant subset of NKT cells expresses an invariant TCR α chain and are therefore referred to as invariant NKT cells (iNKT) [46,47]. Functionally distinct NKT cells subsets can either suppress inflammation in the pathological context of autoimmunity and allograft rejection or promote inflammation in the pathological context of tumor and infection [47,48]. A small subset of CD4+CD8+ double positive (DP) thymocytes are NKT precursors, which give rise to CD44 ^low^ NK1.1 immature iNKT cells and subsequently CD44 ^high^ NK1.1 mature iNKT cells. While several transcriptional factors including retinoid orphan receptor, promyelocytic leukemia zinc finger (PLZF), NF-κB, and T-box expressed in T-cells (T-bet) regulate the initiation, differentiation and maturation of iNKT, Myc protein is also considered to be an essential factor in iNKT development in the thymus [49,50]. Myc protein is highly expressed in CD44 ^low^ NK1.1 DP thymocytes, whereas the expression level of MYC in CD44 ^high^ NK1.1 is intermediate, suggesting that myc protein is upregulated during the initiation and proliferation stages and then is stabilized at intermediate levels at the late development stage [51]. The ablation of MYC at the CD4+CD8+ DP stage leads to a progressive reduction of iNKT cells in mice. This is largely due to the suppression of proliferation instead of inducing apoptosis. In addition, MYC deficient iNKT CD44 ^low^ NK1.1and CD44 ^high^ NK1.1 cells display a reduced production of IL-4, which is a key cytokine required for the development of iNKT cells [49,51].

### 2.4 Dendritic Cells

Dendritic cells (DCs) are bone marrow derived innate immune cells that are distributed in all tissues. DCs are professional antigen presenting cells (APCs) that are capable of capturing and presenting antigen in the form of peptide–major histocompatibility complex (MHC) molecule complexes. Immature DCs, once activated, migrate from the blood to the lymph nodes where they direct adaptive immune response by directly interacting with T and B cells [52,53,54]. The hematopoietic progenitor cells display a high expression level of two Myc family members, MYC and MYCN, whereas the lineage engagement of DCs from progenitor cells leads to a dramatic reduction of these two genes. By contrast, the third Myc family member, MYCL is preferentially expressed in DCs at various development stages [55]. The pro-inflammatory cytokines granulocyte-macrophage colony-stimulating factor (GM-CSF), Interferon-β (IFN- β), and IFN-γ, which are required for maintaining DCs homeostasis, play instrumental roles in regulating MYCL expression in DCs. Mechanistically, transcription factor Interferon regulatory factor 8 (IRF8) directly binds to MYCL promoter and drives its expression in myeloid progenitors and DCs [55,56]. While the development of all DC subsets is largely unperturbed in the absence of MYCL, MYCL deficiency leads to the reduction of the total number and percentage of DCs in lymphoid and peripheral tissues, implicating an essential role of MYCL in maintaining the homeostasis of DCs [55]. In addition to its role in regulating DC proliferation and cell death, MYCL is also involved in regulating T cell priming, a key function of DCs. As such, loss of MYCL significantly decreases in vivo T cell priming following Listeria monocytogenes and vesicular stomatitis infection [55,56]. 

### 2.5 Macrophages

Macrophages, similar to DCs, are considered first-line effectors of innate immunity and largely derived from monocytes. Monocytes that are derived from a myeloid progenitor in the bone marrow can migrate to peripheral blood and are further distributed throughout the body, where they differentiate into tissue macrophage [57]. Macrophages can directly engulf pathogens and apoptotic cells and present antigens and produce immune effectors. They are key players in maintaining the tissue homeostasis, shaping adaptive immune response, inflammation, and tissue repair [58,59,60]. In response to signals from the local microenvironment, macrophages are polarized into distinct phenotypic subtypes, which are generally referred to as classically activated macrophages (M1) and alternatively activated macrophages (M2). The M1 macrophages are proinflammatory cells and are induced by a combination of bacterial product lipopolysaccharide (LPS) and proinflammatory cytokine Interferon (IFN)-γ [61], whereas the polarization of macrophages via the alternative activation program that is triggered by exposure to IL-4 or IL-13 lead to anti-inflammatory M2 subtype. In addition, macrophages that reside within a tumor are often referred to as tumor-associated macrophages (TAMs), which often display M2-like phenotypes with wound healing or immune regulatory functions to support tumor development [62,63,64]. 

Colony-stimulating factor-1 (CSF-1), a mitogenic stimulator of macrophage and its progenitor cells, can induce myc protein expression and drive the survival, proliferation, and maturation of macrophages [65,66,67]. Additionally, M2-polarizing condition induces myc protein expression and nucleus translocation [68]. By contrast, pro-inflammatory stimulation with LPS and IFN-γ suppresses myc protein expression and proliferation in macrophages [65,69,70]. Interestingly, MYCN has also been recently revealed as the most highly upregulated gene in macrophages upon the treatment of immune suppressive soluble factors that are released from apoptotic cells [71]. These studies indicate that MYC and MYCN may not only regulate proliferation but also exert immune modulatory functions in macrophages. Particularly, MYC coordinates with IL-4 downstream signaling mediators, signal transducer and activator of transcription-6 (STAT6) and peroxisome proliferator-activated receptor γ (PPARγ), to direct the expression of a major subset of genes associated with an alternative activation program [68]. While deletion of MYC in the myeloid compartment does not result in alterations in terms of cell number or distribution of macrophages and their progenitors in steady-state, deletion of MYC in macrophages attenuates the pro-tumor function of TAM and suppresses tumor growth [72]. Moreover, germline deletion of MYC results in embryonic death largely due to the defects of vascular endothelial growth factor (VEGF) dependent vasculogenesis [73]. Consistent with the role of TAMs in driving angiogenesis and vascular remodeling, deletion of MYC in macrophage blocks the expression of VEGF and other pro-angiogenic molecules including matrix metalloproteinases-9 (MMP9), hypoxia-inducible factor-1α (HIF-1α), and transforming growth factor- β (TGF-β), collectively leading to the impairment of tumor angiogenesis [72,73]. These studies implicate MYC as a key player in regulating macrophage functions and suggest that MYC inactivation may suppress tumor growth in a cancer cell-extrinsic manner.

## 3. MYC in Regulating Immune Metabolism

**Figure 2:**
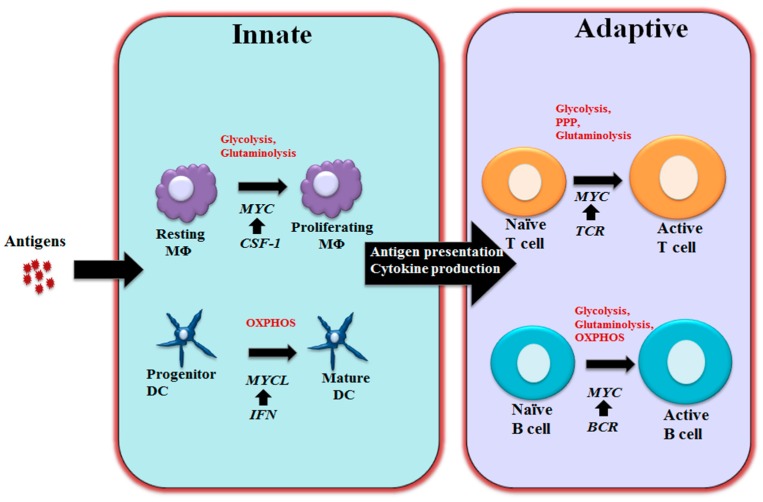
MYC-dependent metabolic reprograming in immunity As schematically shown here, MYC regulates immune cell metabolic reprogramming in different contexts. Colony-stimulating factor-1 (CSF-1) induces MYC expression and engages glycolysis and glutaminolysis that are required for driving macrophage proliferation. Maturation of DCs relies oxidative phosphorylation (OXPHOS) and is associated with Interferon (IFN) mediated MYCL upregulation. MYC is a component of the T cell receptor (TCR) mediated transcriptional network in T cells and coordinately controls glycolysis, pentose phosphate pathway (PPP) and glutaminolysis to support proliferation and differentiation following T cell activation. Upon BCR stimulation, upregulated MYC drives glycolysis, glutaminolysis and OXPHOS to support B cell growth and proliferation. MΦ: Macrophages; DC: Dendritic cells; BCR: B cell receptor.

The shift from glucose oxidation toward aerobic glycolysis (i.e., the Warburg Effect) and heightened glutamine oxidation are characteristic hallmarks of cancer cells [74,75]. The consumption of glucose through aerobic glycolysis is a less efficient, but much faster, way to generate ATP than mitochondrial-dependent oxidative phosphorylation. Glutamine oxidation is often referred to as “glutaminolysis” by analogy to glycolysis [76]. In this process, glutamine is converted to glutamate, and subsequently to the anaplerotic substrate of the tricarboxylic acid (TCA) cycle, α-ketoglutarate (α-KG), which further fuels mitochondrial ATP production [76]. Glucose and glutamine are also important carbon and nitrogen donors that are required for the biosynthesis of amino acids, nucleotides, polyamines and lipids, all of which are the building blocks for supporting cell growth and proliferation. The metabolic rewiring of cancer cells relies on a hierarchical oncogenic cascade involved in Akt/mammalian target of rapamycin (mTOR), mitogen-activated protein kinase (MAPK) signaling, and, essentially, a MYC dependent metabolic transcriptome [77,78]. The evolution of vertebrate immunity has culminated in an effective system that requires the strict coordination of a metabolic program with immune cell development and functionalities. The invading pathogens of vertebrates often rapidly expand and spread throughout the host, requiring immune cells to exert a robust immune response under diverse tissue microenvironment with fluctuations in environmental nutrient levels. As such, the cells of the immune system are constantly exposed to environmental challenges and are capable of tailoring their metabolic programs to meet distinct physiological needs. MYC is one of the key players that coordinate metabolic reprogramming and immune function in immune cells (Figure 2).

### 3.1 MYC in Regulating T and B Cell Metabolism

Upon stimulation of antigen receptors, quiescent naive T cells undergo a 1-day growth phase, followed by massive proliferation, differentiation, and migration [79,80]. T cell metabolic pathways are tightly and ubiquitously linked with T cell activation, differentiation, and immune functions [81,82,83,84,85,86,87,88,89]. Upon activation, a metabolic reprogramming is required for directing nutrients to fuel the biosynthesis of biomass building blocks, which prepare T cells for immune defense and regulation [90,91]. Essentially, naive T cells rapidly switch from fatty acid oxidation and mitochondrial-dependent oxidative phosphorylation of glucose (OXPHOS) to robust aerobic glycolysis and glutaminolysis, priming the activated T cells for the subsequent proliferative burst and differentiation. This change is reminiscent of the characteristic metabolic switch from respiration to aerobic glycolysis (i.e., the Warburg Effect) and glutaminolysis that occurs during cellular transformation to tumor tissue [74]. Like the metabolic regulation in cancer cells, the change in T cell metabolism relies on a hierarchical signaling cascade involving PI3K/Akt, AMP-activated protein kinase (AMPK)/mTOR and MAPK [89,92,93,94,95,96,97]. Meanwhile, lipid metabolism is under the dynamic regulation of nuclear receptor- liver X Receptor (LXR) and the orphan steroid receptor, Estrogen-related receptor alpha (ERRα) [82,83,84,98,99,100,101]. 

MYC plays a critical role in regulating metabolic reprogramming and drives a metabolic transcriptome that precedes cell cycle entry in T cells upon antigen stimulation. The transcription of metabolic enzymes and transporters involved in almost every step of glycolysis are upregulated in a MYC-dependent manner following T cell activation. For glutaminolysis in T cells, MYC is required for driving the transcription of key glutamine transporters and enzymes, including solute carrier family 32a1 (SLC32a1), SLC32a2, and glutaminase 2 (GLS2). Although recent studies indicated that the tumor suppressor p53 is required for the induction of GLS2 in human tumor cells [102,103], the upregulation of GLS2 in murine T cells is a p53-independent event [104]. The fact that MYC controls glutamine uptake and catabolism through transcription of SLC3a2, SLC5A1, SLC7A1, and GLS1 in tumor cells [105,106] indicates that additional signaling events are involved in determining the target preference of MYC between tumor cells and activated T cells. Glutamine is an important nitrogen- and carbon-donor for a variety of biosynthetic precursors including polyamines, a group of essential metabolites that are required for cell proliferation and other cellular processes [107,108,109]. Overexpression of MYC in transformed cell lines induces the expression of ornithine decarboxylase (ODC) that converts ornithine to polyamines, [110]. In T cells, antigen stimulation triggers a MYC-dependent induction of glutaminolysis, which couples with a noncanonical, but MYC-dependent polyamine biosynthetic pathway upon activation. Metabolic enzymes including aldehyde dehydrogenase 18 family member A1 (ALDH18A1), proline dehydrogenase (PRODH), and ornithine aminotransferase (OAT), which links glutamine and proline to ornithine and are induced in a MYC-dependent manner during T cell activation, leading to a massive increase in polyamine synthesis [104].

In response to pathogen challenge or tissue damage, CD4+ T cells also undergo lineage differentiation, the deregulation of which accounts for the pathogenesis of various immunological diseases [111,112]. T helper type 17 (T_H_17) cells and induced regulatory T (iT_reg_) cells, two closely related subsets [113,114,115], differentially utilize glucose and fatty acids as their primary fuels, respectively [86,94,116]. MYC has been identified as one of the core transcriptional factors for T_H_17 differentiation, and a polymorphism close to the MYC genomic locus has been linked to multiple sclerosis susceptibility in human patients [117,118]. Moreover, pharmacologic inhibition of MYC or its upstream regulator, BET bromo domain, suppresses T_H_17-mediated pathology in mouse models [119,120]. T helper type 2 (T_H_2) cell is another key CD4+ T cell lineage, which plays a key role in host immune defense against parasites and contributes to allergy and hypersensitivity. MYC as a key downstream effector of the mTOR pathway controls glucose metabolism and directs T_H_2 cell differentiation [121].

The heightened aerobic glycolysis has also been implicated as a key metabolic feature of B cells following LPS stimulation or the engagement of BCR [122]. Unlike the metabolic reprogramming in T cells following activation, which displays a reciprocal upregulation of glycolysis and suppression of oxygen consumption, B cells display a balanced induction of glycolysis and oxygen consumption following activation, which correlate with a proportionally increased glucose transporter 1 (Glut1) expression and mitochondrial mass [122]. Induction of glycolysis is required for driving antibody (Ab) production, as the inhibition of glycolysis by pharmacological inhibitor not only suppresses active B cell proliferation but also inhibits Ab secretion in vitro and in vivo [122]. While the heightened glycolysis during B cell development in bone marrow is under the control of HIF1α [123], activation of B cells drives a MYC-dependent upregulation of Glut1 and glycolysis. Similar to T cells, glutamine oxidation is also induced in a MYC-dependent manner in B cells. However, the heightened pyruvate oxidation that contributes to oxygen consumption is independent of MYC expression [122]. Although other signaling pathways contribute to B cell metabolic reprogramming [124,125,126], these studies demonstrate that MYC plays a key role in the initiation of metabolic reprogramming in active B cells (Figure 2).

### 3.2 MYC in Regulating Macrophage and Dendritic Cells Metabolism

As the crucial front-line of defense against pathogens, macrophages constantly change their physiology in response to environmental cues. Mitogenic stimulations drive quiescent macrophages entering into the cell cycle, enhancing glycolysis and glutaminolysis to support cell growth. Pro-inflammatory stimulations, however, attenuate cell proliferation, while still engaging glycolysis and pentose phosphate pathway (PPP) to support the production of bactericidal factors. The inability to accommodate these demands would result in homeostatic imbalances in macrophages [64,91,127,128,129,130]. The heightened glycolysis and glutaminolysis in proliferating macrophage is reminiscent of metabolic features in tumor cells, where metabolic reprogramming is driven by aberrant MYC signaling [131,132,133]. Similarly, the MYC-dependent transcriptional program is not only responsible for cell cycle entry, but also drives the upregulation of glucose and glutamine catabolism in macrophages upon mitogenic stimulation. However, pro-inflammatory stimulation suppresses MYC-dependent cell proliferation while engaging a HIF1α-dependent transcriptional program to maintain the heightened glycolysis in M1 macrophages [65,134,135,136]. The switch between the MYC and HIF1α-dependent transcriptional programs may ensure inflammatory M1 macrophages have sufficient metabolic capacity to support their pro-inflammatory functions, while sparing bioenergetic consumptions associated with cell proliferation. Similar to M1 macrophages, activation of DCs by a range of pro-inflammatory stimuli including LPS, the Toll-like receptor -3 (TLR3) ligand poly (I:C), and type I IFN), rewires their metabolic program from mitochondrial oxidative phosphorylation to glycolysis and PPP [127,137]. PPP generates Nicotinamide adenine dinucleotide phosphate (NADPH), which is essential for redox balance, while glycolysis ensures ATP production when mitochondrial oxidative phosphorylation is inhibited by nitric oxide (NO) [135,138,139,140,141]. NADH dehydrogenase (ubiquinone) Fe-S protein 5 (NDUFS5), a key component in complex I of the electron transport chain (ETC), is diminished in MYCL deficient DCs. However, the role of MYCL in regulating DC metabolism remains to be explored [55,137].

## 4. Conclusions

Accumulating evidence has demonstrated that MYC is a key regulator of many fundamental cellular processes in immune cells, most prominently of metabolic programs. Recent findings have revealed that MYC binds to the promoter region of a broad spectrum of active genes to further promote their expression, identifying MYC as a global transcriptional amplifier [44,142]. However, little is known about how MYC achieves functional diversity under different physio-pathological contexts in a range of cell types. Clearly, it will be important to fully dissect the dynamic interplays between MYC and other key cell signaling modulators in different cellular and immune contexts. The growing interests in MYC and metabolism in the immune system will improve our fundamental understanding of the biology of the immune system. In addition, new strategies that target MYC or its downstream metabolic programs will not only advance the treatment of immunological diseases, but will also hold the promise of impacting immunotherapies in treating cancer and other diseases.
